# Alterations in endogenous progesterone metabolism associated with spontaneous very preterm delivery

**DOI:** 10.1093/hropen/hoaa007

**Published:** 2020-04-06

**Authors:** Avinash S Patil, Nilesh W Gaikwad, Chad A Grotegut, Shelley D Dowden, David M Haas

**Affiliations:** 1 Division of Clinical Pharmacology, Department of Medicine, Indiana University School of Medicine, Indianapolis, IN, USA; 2 Department of Obstetrics and Gynecology, Indiana University School of Medicine, Indianapolis, IN, USA; 3 Department of Obstetrics and Gynecology, University of Arizona College of Medicine-Phoenix, Phoenix, AZ, USA; 4 Department of Obstetrics and Gynecology, Creighton University School of Medicine-PRC, Phoenix, AZ, USA; 5 Valley Perinatal Services, Phoenix, AZ, USA; 6 Gaikwad Steroidomics Laboratory, Davis, CA, USA; 7 Department of Obstetrics and Gynecology, Duke University, Durham, NC, USA

**Keywords:** preterm delivery, prematurity, biomarker, steroids, progesterone, 11-deoxycorticosterone, 16-alpha-hydroxyprogesterone, pregnancy

## Abstract

**STUDY QUESTION:**

Do maternal serum levels of progesterone metabolites early in pregnancy correspond to an increased risk for very preterm delivery prior to 32 weeks?

**SUMMARY ANSWER:**

Maternal serum levels of 11-deoxycorticosterone (DOC) measured during the late first trimester or early second trimester correlate with an increased risk for preterm delivery prior to 32 weeks, and the correlation becomes stronger when the ratio of DOC to 16-alpha-hydroxyprogesterone was measured.

**WHAT IS KNOWN ALREADY:**

Progesterone is a pro-gestational steroid hormone that has been shown to decrease the risk of preterm birth in some pregnant women. Progesterone is metabolized by the body into various metabolites including members of the mineralocorticoid and glucocorticoid families. Our group has previously demonstrated that some progesterone metabolites enhance myometrial contractility in an *ex vivo* system, while others result in myometrial relaxation. The current exploratory study was designed to determine if pre-specified metabolites of progesterone measured early in pregnancy were associated with a woman’s risk for delivery prior to 32 weeks, which is referred to as a very preterm delivery.

**STUDY DESIGN, SIZE, DURATION:**

The Building Blocks of Pregnancy Biobank (BBPB) is a biorepository at Indiana University (IU) that follows women prospectively through their pregnancy. A variety of biospecimens are collected at various time points during a woman’s pregnancy. Women participating in the IU BBPB who were enrolled after 8 weeks’ gestation with pregnancy outcome data were eligible for participation.

**PARTICIPANTS/MATERIALS, SETTING, METHODS:**

Women delivering prior to 37 weeks (preterm) and at or after 37 weeks (term) who had blood samples collected during the late first trimester/early second trimester and/or during the early third trimester were identified. These samples were then processed for mass spectroscopy, and the amount of progesterone and progesterone metabolites in the samples were measured. Mean values of each measured steroid metabolite were calculated and compared among women delivering at less than 32 weeks, less than 37 weeks and greater than or equal to 37 weeks. Receiver operating characteristic (ROC) curves were constructed and threshold levels determined for each compound to identify a level above or below which best predicted a woman’s risk for delivery prior to 32 and prior to 37 weeks. Mann–Whitney *U* nonparametric testing with Holm–Bonferroni correction for multiple comparisons was utilized to identify steroid ratios that could differentiate women delivering spontaneously at less than 32 weeks from all other pregnancies.

**MAIN RESULTS AND THE ROLE OF CHANCE:**

Steroid hormone levels and pregnancy outcome data were available for 93 women; 28 delivering prior to 32 weeks, 40 delivering between 32 0/7 and 36 6/7 weeks and 25 delivering at or greater than 37 weeks: the mean gestational age at delivery within the three groups was 27.0, 34.4 and 38.8 weeks, respectively. Among women delivering spontaneously at less than 37 weeks, maternal 11-deoxycorticosterone (DOC) levels drawn in the late first trimester/early second trimester were significantly associated with spontaneous preterm delivery prior to 32 weeks; a threshold level of 47.5 pg/ml had 78% sensitivity, 73% specificity and an AUC of 0.77 (*P* = 0.044). When DOC levels were analyzed as a ratio with other measured steroid hormones, the ratio of DOC to 16-alpha-hydroxyprogesterone among women delivering spontaneously prior to 37 weeks was able to significantly discriminate women delivering prior to 32 weeks from those delivering at or greater than 32 weeks, with a threshold value of 0.2 with 89% sensitivity, 91% specificity and an AUC of 0.92 (*P* = 0.002). When the entire study cohort population was considered, including women delivering at term and women having an iatrogenic preterm delivery, the ratio of DOC to 16-alpha-hydroxyprogesterone was able to discriminate women delivering spontaneously prior to 32 weeks from the rest of the population at a threshold of 0.18 and 89% sensitivity, 59% specificity and an AUC of 0.81 (*P* = 0.003).

**LIMITATIONS, REASONS FOR CAUTION:**

This is a discovery study, and the findings have not been validated on an independent cohort. To mitigate issues with multiple comparisons, we limited our study to pre-specified metabolites that are most representative of the major metabolic pathways for progesterone, and adjustments for multiple comparisons were made.

**WIDER IMPLICATIONS OF THE FINDINGS:**

Spontaneous preterm birth is increasingly being recognized to represent a common end pathway for a number of different disease phenotypes that include infection, inflammation, premature rupture of the membranes, uterine over distension, cervical insufficiency, placental dysfunction and genetic predisposition. In addition to these phenotypes, longitudinal changes in the maternal–fetal hypothalamic–pituitary–adrenal (HPA) axis also likely contribute to a significant proportion of the disease burden of spontaneous preterm birth. Here, we demonstrate that differential production of steroid metabolites is associated with very early preterm birth. The identified biomarkers may hint at a pathophysiologic mechanism and changes in the maternal–fetal dyad that result in preterm delivery. The early identification of abnormal changes in HPA axis metabolites may allow for targeted interventions that reverse the aberrant steroid metabolic profile to a more favorable one, thereby decreasing the risk for early delivery. Further research is therefore required to validate and extend the results presented here.

**STUDY FUNDING/COMPETING INTEREST(S):**

Funding for this study was provided from the Office of the Vice Chancellor for Research at IUPUI, ‘Funding Opportunities for Research Commercialization and Economic Success (FORCES) grant’.

Both A.S.P. and C.A.G. are affiliated with Nixxi, a biotech startup. The remaining authors report no conflict of interest.

**TRIAL REGISTRATION NUMBER:**

Not applicable.

**WHAT DOES THIS MEAN FOR PATIENTS?**

Being born before 32 weeks (preterm birth is delivery less than 37 weeks) can cause a wide number of long-term health issues for children or even the death of newborn babies. At the moment, doctors do not have many ways of helping them know which pregnant women will deliver their babies prematurely. Being able to know which women might have a preterm delivery is important so that various treatments could be used to decrease the chance of these women delivering early.

In this study, the authors show that measurement of two different steroid hormones in the blood of women early in pregnancy might be able to predict which women will deliver prior to 32 weeks, which is also called *very preterm delivery*. The authors intend to do more research to find out if treating women who are thought to be likely to deliver before term can make delivering early less likely.

## Introduction

Preterm birth continues to be a significant global health problem. Approximately 1 in 10 pregnant women deliver prematurely in the USA, with worldwide rates rising up to 18% (World Health Organization, 2019). Preterm birth has widespread societal impact: newborns can have lifelong medical complications, families deal with emotional strain and financial costs rise for health plans and employers. Among babies born prematurely, those with very or extremely preterm delivery (less than 32 weeks’ and 28 weeks’ gestation, respectively) have the most severe complications and highest associated healthcare costs annually (World Health Organization, 2019). In the USA, preterm delivery less than 32 weeks is associated with an 80-fold increase in infant mortality compared to women delivering between 39 and 41 weeks’ gestation (175.5 versus 2.1 deaths per 1000 live births) (Mathews and MacDorman, 2012). Despite technological advances, the rate of very preterm delivery (less than 32 weeks’ gestation) remains unchanged (Heron, 2012). Across the USA, the annual societal economic costs of preterm birth and associated care are estimated at $26 billion (Behrman and Butler, 2007). The lack of progress in reducing the rate of early preterm delivery has hampered efforts to improve downstream sequelae, including disparities in infant mortality rates.

One barrier to the reduction of spontaneous preterm delivery (sPTD) less than 32 weeks is the development of an accurate risk stratification approach for women early in pregnancy. The current standard of care is to identify women at risk for sPTD based on a history of premature delivery in a prior pregnancy. However, this approach has been estimated to miss up to 93% of sPTD annually (Khalifeh and Berghella, 2016). A fundamental challenge to risk stratification is that the pathogenesis of preterm delivery is multifactorial. A recent multicenter analysis identified nine potential phenotypes associated with the common final pathway of uterine contractility and sPTD (Manuck *et al.*, 2015). The diversity of etiologies of preterm delivery presents a barrier for any single testing approach to identify all women at risk of preterm delivery.

A long-standing phenotype associated with sPTD involves the maternal/fetal hypothalamic–pituitary–adrenal (HPA) axis. Central to this phenotype is the role of corticotropin-releasing hormone (CRH) in leading to a cascade of events culminating in delivery (McLean *et al.*, 1995; McGrath *et al.*, 2002; Sandman *et al.,* 2006). The production of placental CRH has been suggested to be an integrative pathway which allows prenatal stressors to influence gestational length (Sandman, 2018). A longitudinal study of 203 pregnancies revealed that elevations in maternal cortisol as early as 15 weeks’ gestation were associated with increased third trimester CRH concentrations and preterm birth rates (Sandman *et al.*, 2006). However, a large secondary analysis did not find that CRH alone was predictive of recurrent preterm delivery when assessed early in pregnancy at 16–20 weeks (Sibai *et al.*, 2005).

Regulation of preterm labor through the HPA axis is subject to hormonal influences. Endogenous steroids, such as glucocorticoids, have been identified as key promoters of placental CRH gene expression (Robinson *et al.*, 1988; Karalis *et al.,* 1996; Whittle *et al.*, 2001; Wang *et al.*, 2013), while progesterone has been suggested to mitigate this effect. Glucocorticoids and progesterone have been associated with opposing influences on uterine contractility (Whittle *et al.,* 2001). Metabolites of progesterone have been shown to have varying efficacy in mitigating uterine contractility, with some metabolites increasing contractility, unlike progesterone (Kubli-Garfias *et al.*, 1979; Patil *et al.*, 2015). Despite the known influence of these molecules on sPTD, there has not been a systematic evaluation of endogenous steroid hormones derived from progesterone. In this exploratory study, we sought to evaluate a range of steroid hormones derived from progesterone with both mineralocorticoid and glucocorticoid characteristics for association with sPTD less than 32 weeks. We hypothesized that multiple steroid hormones may influence sPTD potentially through modulation of placental CRH.

## Materials and Methods

In order to investigate the relationship between endogenous steroid hormones and the timing of preterm birth, metabolomic analysis was performed on prospectively collected plasma specimens from a cohort of women enrolled in a single-institution pregnancy registry. The Building Blocks of Pregnancy Biobank at the Indiana University School of Medicine (IU BBPB) is a longitudinal, prospective pregnancy biorepository with a range of maternal specimens collected as part of an organized research program. Subjects enrolled in the IU BBPB have biologic specimens collected through convenience sampling every trimester over the course of their pregnancy, in addition to clinical data on current and prior obstetric outcomes. Use of biological specimens and associated data was covered by existing Institute Review Board approval for the IU BBPB (IRB #1011003384).

**Table I:** Demographic data for all 93 subjects in the study.

	**GA at delivery (weeks)**		
**Parameter**	Less than 32	32 0/7–36 6/7	Greater than or equal to 37 0/7	***P* value**
*N (%)*	28 (30%)	40 (43%)	25 (27%)	
Age (years)	25.5	27.6	23.6	0.04
GA at delivery (weeks, mean)	27	34.4	38.8	<0.001
BMI (kg/m^2^)	32	32	27	0.06
History of PTD, %	36%	43%	13%	0.51
Race^1^ (%)				0.45
*Caucasian*	13 (46%)	23 (57%)	14 (56%)	
*African American*	14 (50%)	17 (43%)	10 (40%)	
*Asian*	0	0	1 (4%)	
*Not reported*	1 (4%)	0	0	
Composite maternal^2^	0.5 (0–1.0)	1.0 (0–2.0)	0 (0–0)	<0.001
Composite antepartum^3^	1.0 (0–1.0)	1.0 (0–2.0)	0 (0–0)	<0.001

^1^Analysis by chi-square; all others by ANOVA.

^2^Median (interquartile range: IQR) number of pre-specified maternal co-morbidities (see methods).

^3^Median (IQR) number of pre-specified antepartum complications (see Materials and methods).

GA = gestation age, PTD = preterm delivery

**Table II:** Demographic data for women experiencing spontaneous PTD at two time points in the enriched cohort.

**Parameter**	**sPTD less than 32 weeks**	**sPTD greater than or equal to 32 weeks**	***P* value**
*N (%)*	17 (50%)	17 (50%)	
Age (years)	24.6	26.6	0.32
GA at delivery (weeks, mean)	26.8	34.4	<0.001
BMI (kg/m^2^)	30.9	29.5	0.58
History of PTD, %	47%	35%	0.73
Race (%)			0.59^1^
*Caucasian*	8 (47%)	9 (53%)	
*African American*	8 (47%)	8 (47%)	
*Not reported*	1 (6%)	0	
Composite maternal^2^	0 (0–1.0)	1.0 (0–2.0)	0.12
Composite antepartum^3^	1.0 (0–1.0)	0 (0–1.0)	0.82

^1^Analysis by chi-square; all others by ANOVA.

^2^Median (IQR) number of pre-specified maternal co-morbidities (see Materials and methods).

^3^Median (IQR) number of pre-specified antepartum complications (see Materials and methods).

sPTD = spontaneous preterm delivery. The enriched cohort experienced sPTD at less than 37 weeks.

We sought to examine the association of sPTD less than 32 weeks with three endogenous steroid pathways: progestogens, glucocorticoids and mineralocorticoids. Steroid molecules were selected for quantification based on their characterization as immediate progesterone derivatives or downstream metabolites with mineralocorticoid or glucocorticoid activity. The steroid molecules chosen for quantification included progesterone, 17-hydroxyprogesterone (17-OHP), 11-deoxycortisol, cortisol, 11-deoxycorticosterone (DOC), 17-deoxycortisol, 20α-dihydroprogesterone, 17α,20α-dihydroxyprogesterone, 16α-hydroxyprogesterone (16α-OHP), 6α-hydroxyprogesterone (6α-OHP) and 6β-hydroxyprogesterone (6β-OHP). Prospectively collected plasma specimens from women enrolled in the IU BBPB were obtained for the analysis. Plasma samples were divided into two epochs for analysis based on when in pregnancy they were collected; Epoch 1 (late first trimester/early second trimester) and Epoch 2 (early third trimester). Specimens were obtained from all available subjects who delivered preterm (less than 37 weeks) for the initial analysis. Specimens from subjects delivered at term (greater than or equal to 37 weeks) were also collected during the same time period and processed identically. Subjects delivered at term were matched for BMI and excluded for use of steroids or anticoagulants. A targeted subset of the steroid molecules was compared between the term and preterm specimens.

A targeted metabolomics approach, developed at Gaikwad Steroidomics Lab, was used to quantify endogenous progestogen, glucocorticoid and mineralocorticoid steroids using ultraperformance liquid chromatography-tandem mass spectrometry (UPLC/MS-MS) analysis. The frozen human plasma samples were processed using an assay validated on the MS platform (Gaikwad, 2013). Briefly, 1–2-ml aliquots of plasma were adjusted to pH 7.0 and subject to solid phase extraction with methanol. The methanol fraction was subjected to UPLC/MS-MS analysis. Analytical separations on the UPLC system were conducted with C18 or phenyl columns (1 × 100 mm) at a flow rate of 0.15 ml/min. The gradient was started with 100% A (0.1% formic acid in H_2_O) and 0% B (0.1% formic acid in CH_3_CN), changed to 80% A over 10 min, followed by a 10-min linear gradient to 0% A, resulting in a total separation time of 20 min. The elutions from the UPLC column were introduced to the mass spectrometer. All MS experiments were performed using electrospray ionization (ESI) in positive ion and negative ion mode, with an ESI-MS capillary voltage of 3.0 kV, an extractor cone voltage of 2 V and a detector voltage of 650 V. The following MS conditions were used: desolvation gas at 600 l/h, cone gas flow at 60 l/h, desolvation temperature at 200°C and source temperature 100°C. Using pure reference standards, the multiple reaction monitoring method (MRM) for UPLC/MS-MS operation was previously generated. Pure standards were used to optimize the UPLC-MS/MS conditions prior to analysis and make calibration curves. Elutions from the UPLC column were analyzed in the MRM mode, and resulting data were processed using MassLynx 4.1 software (Waters Corporation, Milford, MA, USA).

Quantified steroids were evaluated for association with sPTD less than 32 weeks using standard statistical methods. Statistical analysis was performed using SPSS Statistics Version 26 software (IBM, Armonk, NY, USA). Receiver operating characteristic (ROC) curves were used to identify steroid hormone patterns capable of distinguishing women who would subsequently have sPTD less than 32 weeks from women who would deliver at or greater than 32 weeks’ gestation (delivery at 32 0/7–36 6/7 weeks or greater than or equal to 37 weeks). The area under the ROC curve (AUROC) was used to compare the predictive ability of individual and combinations of steroid hormones in Epochs 1 and 2. The Mann–Whitney *U* test with Holm–Bonferroni correction for multiple comparisons was utilized to determine if there was a significant difference in median values of steroid ratios. The initial screening of steroid hormones was performed using an ‘enriched cohort’ of only subjects with sPTD less than 37 weeks (comparison of sPTD less than 32 weeks compared to sPTD in the 32 0/7–36 6/7 weeks cohort). Subsequent analyses also included subjects with iatrogenic PTD and full-term delivery (greater than or equal to 37 weeks’ gestation) to assess performance of the molecules in a generalized population (comparison of sPTD less than 32 weeks versus all other subjects). Patient demographic and clinical outcome data were analyzed using standard statistical approaches. A *P* value <0.05 was considered significant. A composite of maternal co-morbidities was pre-specified for analysis between cohorts: thyroid disorder, diabetes, respiratory disorder, hypertension, seizure disorder, cardiac disease, renal disease, hematologic disease, autoimmune disease, liver disease, cancer, mental health disorder, endocrine disorder, neurologic disorder and infectious diseases. Similarly, a composite of antepartum complications was pre-specified for analysis between cohorts: gestational hypertension, pre-eclampsia, HELLP (hemolysis, elevated liver enzyme, low platelet count) syndrome, gestational diabetes, incompetent cervix, hyperemesis, peripartum depression, oligohydramnios/polyhydramnios, fetal demise, growth restriction, placentation disorders and hydrops.

**Table III:** Analysis of individual steroids by GA within the enriched cohort of women with sPTD at less than 37 weeks and ability to predict sPTD at less than 32 weeks in the enriched cohort.

**Steroid hormone**		**Steroid concentration (pg/ml)** ^**1**^		**ROC curve**	
		**sPTD less than 32 weeks**	**sPTD greater than or equal to 32 weeks**	**AUC (95% CI)**	***P* value**
Progesterone	Epoch 1	5546 ± 9726	945.8 ± 1274.6	0.288 (0.046–0.530)	0.111
	Epoch 2	652.5 ± 277.9	6284 ± 12057.8	0.8 (0.593–1.00)	0.050
17-Hydroxyprogesterone	Epoch 1	901.1 ± 592.3	981.3 ± 1012.3	0.485 (0.214–0.756)	0.909
	Epoch 2	1273.6 ± 535.7	988 ± 758.6	0.293 (0.068–0.519)	0.176
11-Deoxycortisol	Epoch 1	616.3 ± 506.8	483.5 ± 463.7	0.434 (0.167–0.701)	0.621
	Epoch 2	1459.2 ± 565	679.9 ± 938.3	0.133 (0–0.294)	**0.016**
Cortisol	Epoch 1	69 217 ± 41 083	61 727 ± 40 916	0.434 (0.176–0.692)	0.621
	Epoch 2	130 686 ± 85 464	77 717 ± 53 034	0.280 (0–0.577)	0.150
11-Deoxycorticosterone	Epoch 1	43.8 ± 64.8	110.3 ± 126.7	0.768 (0.551–0.984)	**0.044**
	Epoch 2	155 ± 84.8	236.1 ± 241.3	0.547 (0.293–0.801)	0.760
17-Deoxycortisol	Epoch 1	1500.1 ± 2203.7	1465 ± 1330	0.591 (0.329–0.853)	0.494
	Epoch 2	3262.2 ± 2080.5	1974.5 ± 2055.8	0.300 (0–0.623)	0.190
20α-Dihydroprogesterone	Epoch 1	3266.3 ± 3228.8	1667.7 ± 1168.8	0.364 (0.090–0.638)	0.305
	Epoch 2	3988.4 ± 2947.4	3388.6 ± 2515.1	0.467 (0.179–0.754)	0.827
17α,20α-Dihydroxyprogesterone	Epoch 1	454.8 ± 338.8	398.1 ± 292.5	0.444 (0.175–0.714)	0.676
	Epoch 2	458.2 ± 233.2	455.3 ± 342.1	0.413 (0.096–0.731)	0.570
16α-Hydroxyprogesterone	Epoch 1	442.7 ± 284.2	411.6 ± 499.7	0.384 (0.130–0.638)	0.382
	Epoch 2	872.6 ± 439.5	681.9 ± 611.7	0.333 (0.057–0.609)	0.275
6α-Hydroxyprogesterone	Epoch 1	815.3 ± 910.4	638.5 ± 384.2	0.545 (0.266–0.825)	0.732
	Epoch 2	1217 ± 322.6	743.6 ± 347	0.160 (0–0.343)	**0.026**
6β-Hydroxyprogesterone	Epoch 1	401.6 ± 606.8	187 ± 272.5	0.444 (0.177–0.712)	0.676
	Epoch 2	1313.2 ± 691.2	658.7 ± 679.1	0.213 (0–0.428)	0.061

^1^Values are mean ± SD.

*P* value is reported for the AUC of the receiver operating characteristic (ROC) curve for each steroid hormone. *P* values in bold indicate significance.

Epoch 1: blood collected in the late first trimester/early second trimester Epoch 2: blood collected in the early third trimester.

## Results

Ninety-three subjects from the BBPB were included in the study. Of these, 68 delivered preterm (less than 37 weeks) and 25 delivered full term (at or greater than 37 weeks). Twenty-eight subjects (41%) within the preterm group delivered in the very preterm period (less than 32 weeks), while the remaining 40 (59%) delivered between 32 0/7 and 36 6/7 weeks. Thirty-four subjects were determined to have had iatrogenic PTD (34/68, 50%). The most common indication for iatrogenic PTD was pre-eclampsia (20/34, 59%), followed by preterm premature rupture of the membranes (6/34, 18%). Samples were obtained from subjects at a mean (± SD): gestational age of 15.5 ± 3.4 weeks in Epoch 1 (*n* = 71), and at 29.6 ± 3.2 weeks in Epoch 2 (*n* = 45).

Patient demographics were similar between the preterm and full-term groups (Table I). There were no differences in race or history of prior PTD between the preterm and full-term groups. Significant variance in maternal age was observed between groups, with the full-term group having younger subjects. Similarly, the full-term group had lower rates of maternal co-morbidities and antepartum complications than either preterm group (Table I). As described in the Materials and Methods, the initial analyses were performed in the ‘enriched cohort’ of subjects with only sPTD less than 37 weeks’ gestation (of the 68 women prior to 37 weeks’ gestation, 34 delivered spontaneously). Subsequent analyses included all subjects (sPTD, iatrogenic PTD and full-term delivery). Table II provides a comparison of demographics between subjects with sPTD less than 32 weeks and greater than or equal to 32 weeks’ gestation in the enriched cohort. Aside from gestational age at delivery, no significant differences were present in the demographics of the two groups.

All of the targeted steroid molecules could be quantified as intended using the validated MS methods. Analysis of individual steroid molecules within the ‘enriched cohort’ of subjects with sPTD less than 37 weeks is described in Table III. In Epoch 1, DOC was identified as the only molecule with a significant AUROC, which was 0.768 (*P* = 0.044) for the association with sPTD less than 32 weeks within the group of women delivering preterm. A threshold 11-deoxycorticosterone concentration of 47.5 pg/ml was associated with 78% sensitivity and 73% specificity for identification of pregnancies at risk for sPTD less than 32 weeks (AUROC 0.768, *P* = 0.044, Table III). Within the sPTD less than 37 weeks ‘enriched cohort’, subjects with 11-deoxycorticosterone values below the same threshold had a mean gestational age of delivery of 27.7 ± 5.6 weeks, while those with values above the threshold delivered at 32.2 ± 3.5 weeks on average (*P* = 0.045). In Epoch 2, 11-deoxycortisol (AUROC 0.133; *P* = 0.016) and 6α-OHP (AUROC 0.160; *P* = 0.026) were each significantly associated with sPTD <32 weeks.

**Figure 1:**
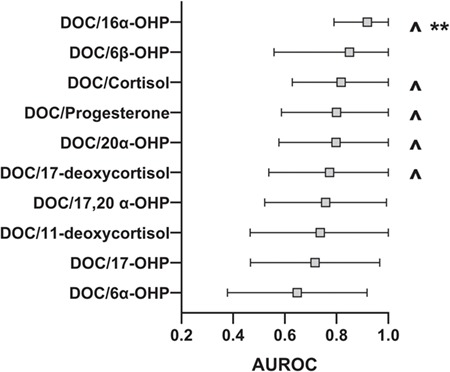
**Prediction of spontaneous preterm delivery less than 32 weeks.** Area under the ROC curve (AUROC with 95% CI) for 11-deoxycorticosterone (DOC) to selected steroid ratios for prediction of spontaneous preterm delivery (sPTD) in the enriched cohort of all sPTDs. DOC steroid ratios with significant predictive ability (ROC curve *P* value) are indicated by a carat symbol (^). Ratios with a significant difference in median values between sPTD less than 32 and sPTD greater than or equal to 32 weeks after correction for multiple comparison (Mann–Whitney *U* nonparametric testing with Holm–Bonferroni correction for multiple comparisons) are indicated by asterisks (^**^). Enriched cohort: comprises only subjects who delivered less than 37 weeks gestation.

DOC was the only steroid to have significant predictive ability in Epoch 1 (AUROC 0.768, *P* = 0.044) and was thus utilized for examination of steroid ratios. Ratios of DOC to the other steroids were assessed to determine if they could provide an improvement in prediction of sPTD less than 32 weeks within Epoch 1 (Fig. 1). Within the enriched cohort of sPTD less than 37 weeks, ROC curve analysis identified DOC/progesterone (AUROC 0.800, *P* = 0.033), DOC/cortisol (AUROC 0.818, *P* = 0.017), DOC/17-deoxycortisol (AUROC 0.773, *P* = 0.048), DOC/20α-dihydroprogesterone (AUROC 0.798, *P* = 0.025) and DOC/16α-hydroxyprogesterone (AUROC 0.919, *P* = 0.002) as meeting statistical significance in Epoch 1 (Fig. 1). The ratio of DOC/16α-OHP, having the highest AUROC, demonstrated 89% sensitivity and 91% specificity for prediction of PTD less than 32 weeks at a threshold value of 0.2. Subjects within the enriched cohort whose DOC/16α-OHP values fell below the threshold had a mean gestational age of delivery of 26.4 ± 4.7 weeks, while those with values above the threshold delivered at 32.8 ± 3.4 weeks (*P* = 0.002). Within Epoch 2, ratios of DOC/11-deoxycortisol (AUROC 0.829, *P* = 0.033) and DOC/6β-hydroxyprogesterone (AUROC 0.817, *P* = 0.045) were significantly associated with sPTD <32 weeks (Fig. 1). Further analysis of the median DOC steroid ratios between sPTD less than 32 and sPTD greater than or equal to 32 week groups in Epochs 1 and 2 was performed using the Mann–Whitney *U* test with Holm–Bonferroni correction to account for multiple comparisons (Fig. 1). The analysis revealed that only the DOC/16α-OHP ratio remained significant (*P* = 0.02 after correction for multiple comparison). Figure 1 illustrates that only the DOC/16α-OHP ratio had both significant predictive characteristics and a significant difference in median values between the sPTD < 32 and sPTD ≥ 32 weeks groups.

**Figure 2:**
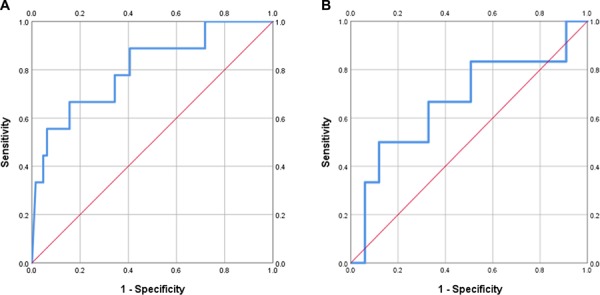
**Assessment of performance of the DOC/16α-hydroxyprogesterone biomarker in the entire study population.** **A** ROC curve of DOC/16α-OHP in Epoch 1 for prediction of sPTD less than 32 weeks from all subjects. AUROC 0.805 (0.644–0.965); *P* = 0.003. **B** ROC curve of DOC/16α-OHP in Epoch 1 for prediction of iatrogenic PTD less than 32 weeks from all subjects. AUROC 0.669 (0.417–0.922); *P* = 0.172. 16α-OHP: 16α-hydroxyprogesterone. Epoch 1: blood collected in the late first trimester/early second trimester. Entire study population: all subjects, including women having an iatrogenic preterm delivery and women delivering at term.

Based on the AUROC within the enriched cohort, the performance of the DOC/16α-OHP biomarker was next assessed in the entire study population, which also included subjects with iatrogenic PTD and full-term deliveries. DOC/16α-OHP was able to successfully predict pregnancies that would result in sPTD less than 32 weeks when measured in the late first trimester/early second trimester in this general population (Fig. 2a: AUROC 0.805; *P* = 0.003). A threshold DOC/16α-OHP ratio of 0.18 was associated with 89% sensitivity, 59% specificity for identification of pregnancies at risk for sPTD less than 32 weeks. The DOC/16α-OHP biomarker did not predict iatrogenic PTD less than 32 weeks (Fig. 2b: AUROC 0.669; *P* = 0.172). Within the entire study population, subjects with a DOC/16α-OHP ratio below the threshold value in Epoch 1 delivered on average at 33 ± 6.1 weeks, while those with a ratio above the threshold delivered at 35.6 ± 4.2 weeks (*P* = 0.04). A similar analysis of DOC alone as a biomarker in the entire study population in Epoch 1 was not associated with a significant difference in gestational ages at birth (30.7 ± 4.8 versus 32 ± 3.7 weeks; *P* = 0.325). Additionally, the Mann–Whitney *U* test confirmed a significant difference in the median DOC/16α-OHP ratio between the sPTD less than 32 week group compared to the remainder of the study population (*P* = 0.003). In Epoch 2, the ratio of DOC/11-deoxycortisol (AUROC 0.738, *P* = 0.019) was predictive of any PTD <32 weeks in the general population. The mean gestational age at delivery for subjects below the threshold value (0.18) for DOC/11-deoxycortisol was 31.7 ± 2.7 weeks compared to 33.8 ± 1.7 weeks for individuals above the threshold value (*P* = 0.007).

## Discussion

Our study demonstrates a relation between progesterone metabolism into the mineralocorticoid and glucocorticoid pathways and timing of sPTD. In the study cohort, DOC measured during the late first trimester/early second trimester was the only steroid hormone evaluated that was able to distinguish subjects with a significantly increased rate of sPTD less than 32 weeks from those delivering spontaneously between 32 0/7 and 36 6/7 weeks’ gestation. The discriminatory ability of this molecule was further improved when its measured concentration was compared to other compounds in Epoch 1, as a ratio. In particular, the ratio of DOC to a number of other molecules (progesterone, cortisol, 17-deoxycortisol, 20α-dihydroprogesterone and 16α-OHP) each resulted in a higher AUROC compared to the AUROC for DOC alone. The ratio of DOC/16α-OHP performed the best (AUROC 0.919), followed by DOC/cortisol (AUROC 0.818). In Epoch 2, the ratio of DOC/11-deoxycortisol showed the best ability to distinguish subjects destined for sPTD less than 32 weeks (AUROC 0.829). The predictive ability of these molecules diminished when they were applied to a general population with outcomes including iatrogenic PTD and full-term deliveries. Nonetheless, the DOC/16α-OHP ratio in Epoch 1 continued to distinguish subjects who would subsequently have sPTD less than 32 weeks with statistical significance (AUROC 0.805, *P* = 0.003) within the entire general population. The DOC/16α-OHP ratio in Epoch 1 was also the only biomarker that met statistical significance for difference in median values between the sPTD < 32 weeks and sPTD ≥32 weeks groups after correction for multiple comparisons (*P* = 0.02).

The goal of this study was to evaluate progesterone-derived molecules in the classic steroid metabolism pathway as candidate predictors of preterm birth. Progesterone is recognized as the hormone primarily responsible for the maintenance of pregnancy through a variety of pro-gestational effects. Progesterone is part of a larger metabolic pathway and is further metabolized by CYP450 21A2 (21-hydroxylase) and CYP450 17A1 (17a-hydroxylase) into a variety of mineralocorticoid and glucocorticoid molecules. While progesterone has beneficial effects for an ongoing pregnancy, the impact of its metabolites can be varied. Patil *et al*. have previously shown that the endogenous progesterone metabolites 16α-OHP and 6β-OHP have opposing effects on oxytocin-induced uterine contraction frequency (Patil *et al.,* 2015). The extent of progesterone metabolism into these opposing compounds is dependent upon cytochrome P450 enzyme isoform, the expression of which may vary between individuals (Quinney *et al.*, 2017). These observations suggest that the ‘progestogen milieu’ may define a subgroup of women with a pro-contractile phenotype who have greater susceptibility to preterm labor when exposed to an additional stimulus (infection, dehydration, trauma, etc.). Similarly, increased concentrations of a glucocorticoid, cortisol, have been associated with preterm labor (Sandman *et al.,* 2006).

**Figure 3:**
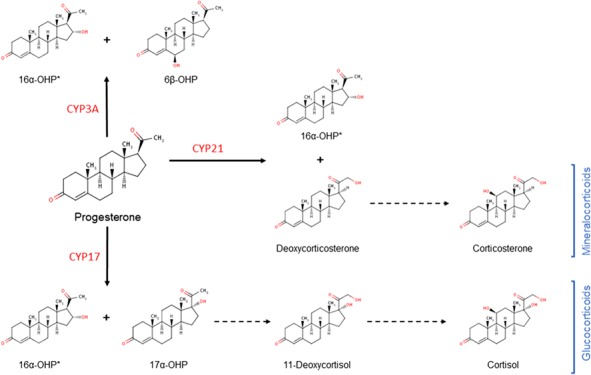
**Progesterone metabolism by major cytochrome P450 enzymes denoting major and minor products.** Asterisk (^*^) denotes minor metabolite of progesterone from the respective enzymatic reactions. CYP: cytochrome P450, 6β-OHP: 6β-hydroxyprogesterone, 17α-OHP: 17-hydroxyprogesterone.

The maternal/fetal HPA phenotype has been extensively studied as a pathway leading to sPTD. The HPA phenotype postulates that CRH influences the timing of delivery (McLean *et al.,* 1995; McGrath *et al.,* 2002; Sandman *et al.*, 2006). CRH is a peptide hormone produced by placental trophoblasts and released into both maternal and fetal compartments. CRH in the maternal circulation binds to CRH binding protein (CRH-BP), which limits its biological effects. However, CRH in the fetal circulation has been shown to bind to CRH receptors (CRH-R1, CRH-R2) in the myometrium, and to stimulate the production of prostaglandins and estrogens (via dehydroepiandrosterone: DHEA), all of which increase uterine contractility. Increased CRH concentrations have been observed as early as 16 weeks and are most prominent shortly before delivery (McLean *et al.*, 1995). The regulatory mechanisms that influence placental CRH production are influenced by endogenous steroids. Glucocorticoids have been identified as a key stimulator of placental CRH (Robinson *et al.,* 1988; Karalis *et al.*, 1996; Whittle *et al.*, 2001; Wang *et al.*, 2013). The rising placental CRH further stimulates fetal adrenal production of cortisol (glucocorticoid), in a positive-feedback loop, which culminates in parturition (Robinson *et al.*, 1988).

Although the association between elevated levels of CRH and timing of preterm birth is established in the literature, identification of women with the HPA phenotype early in gestation has been elusive. Production of maternal plasma CRH occurs along a higher trajectory in women who have sPTD compared to those with term delivery (McGrath *et al.,* 2002). A study by Hill *et al*. identified white blood cell count and maternal CRH as the most accurate biomarkers for preterm birth within 48 h at less than 28 and greater than 28 weeks, respectively (Hill *et al.,* 2008). Notably, cortisol was included in this analysis but did not outperform the other biomarkers. Another study supported the utility of CRH as a biomarker of preterm birth in underrepresented ethnic groups when drawn at 22 to 24 weeks’ gestation (Ruiz *et al.*, 2016). In contrast, a secondary analysis of a preterm birth prevention study performed by the *Eunice Kennedy Shriver* National Institute of Child Health and Human Development Maternal-Fetal Medicine Units Network did not find that CRH alone was predictive of recurrent preterm delivery when assessed at 16 to 20 weeks (Sibai *et al.*, 2005). A longitudinal study of 203 pregnancies revealed that elevations in maternal cortisol as early as 15 weeks’ gestation were associated with increased third trimester CRH concentrations and preterm birth rates (Sandman *et al.,* 2006). Another study found a trend of decreased progesterone and increased CRH in maternal plasma in the early third trimester to be associated with preterm birth (Stamatelou *et al*., 2009). Despite the known role of glucocorticoids as a promoter of CRH, there has not been a broader evaluation of the glucocorticoid class of molecules as predictors of preterm birth.

Of the steroid biomarkers identified in our study, DOC is a metabolite of progesterone with partial mineralocorticoid properties. Notably, it has been observed to be increased in the presence of DHEA-sulfate, which is associated with increased CRH. 16α-OHP is a minor product that serves as a marker for the extent of progesterone metabolism, and 11-deoxycortisol is a precursor to the glucocorticoid cortisol. Our data suggest that a decreased ratio of (DOC/16α-OHP) is an early indicator of an imbalance between mineralocorticoid and glucocorticoid pathways later in pregnancy, and subsequent preterm delivery. 16α-OHP has been documented to be a minor product of progesterone metabolism by cytochrome P450 enzymes, including CYP3A, CYP17 and CYP21 (Fig. 3) (Mizrachi *et al.*, 2011; Storbeck *et al.,* 2011; Quinney *et al.,* 2017). 16α-OHP is unique in that it is not extensively metabolized, while most other progesterone derivatives continue to be modified into mineralocorticoids, glucocorticoids, estrogens or androgens. As a result, 16α-OHP may serve as a marker of progesterone metabolism downstream and the studied DOC/16α-OHP ratio may identify those women with enhanced progesterone metabolism and, as such, an increased risk for preterm birth. The presence of DOC, the major product of CYP21 metabolism of progesterone, as a component of the predictive algorithm suggests that progesterone metabolism into pathways other than glucocorticoids is an important feature that characterizes risk of preterm delivery. Notably, metabolism of progesterone by other major CYP enzymes produces pro-contractile molecules. Progesterone metabolism by CYP3A produces 6β-OHP, which increases uterine contractile frequency (Patil *et al.,* 2015). CYP17 metabolism of progesterone leads to the production of cortisol, which is known to stimulate placental CRH and uterine contractility as part of the maternal–fetal HPA axis phenotype.

The high societal costs associated with preterm birth have driven the development of new approaches for diagnosis and treatment. Prevention of a single very preterm delivery can account for the cost of care for 40 full-term infants over the first year of life. Currently, screening for risk of preterm delivery is largely based upon personal history of preterm delivery in a prior pregnancy. However, many women are not able to be screened because they lack prior obstetric history: 40% of pregnancies occur in nulliparous women (first time pregnancies) where no prior obstetric history exists (Martin *et al.*, 2013). Also notable is that 70% of pregnant women delivering prematurely in their second pregnancy had full-term deliveries in their first pregnancy (Laughon *et al.*, 2014). In recent years, a variety of ‘-omics’ technologies (proteomics, genomics) have been used to develop laboratory-based methods for the prediction of sPTD (Saade *et al.*, 2016; Jelliffe-Pawlowski *et al.,* 2018; Ngo *et al.*, 2018; McElrath *et al.,* 2019). While these tests provide advances in risk stratification, the mechanistic relationship of their biomarkers to the onset of preterm labor is not well established. The diversity of clinical phenotypes for preterm birth undermines the plausibility of any single test providing a comprehensive risk assessment early in pregnancy before the phenotype is evident; rather, new tests should be designed to characterize a sub-population of women with a single phenotype early in pregnancy that may portend an increased risk of preterm delivery and provide insight into appropriate interventions.

Our study has several limitations that we acknowledge. First, the study was designed as an exploratory analysis of a pre-specified range of molecules and is subject to multiple-comparisons error. We have sought to minimize this risk by using the Holm–Bonferroni correction to reduce false-positive results and correlating findings to supporting data in the literature. Our study was not powered to directly compare various biomarker combinations; instead, the ROC curve for each biomarker was compared to the line of no-discrimination (random guessing). A future study with a pre-planned hypothesis is needed to confirm the observed associations. Second, our sample size was limited and generalization of the study findings should be made with caution. We utilized an established academic pregnancy biobank to create an enriched study population to increase the feasibility of this discovery study. The 28 very preterm (<32 weeks) births in our enriched cohort is roughly equivalent to the number expected from screening a general population of 1400 pregnancies (1:50 incidence). We applied our findings from the analysis of the enriched cohort to all subjects in our study (including iatrogenic PTD and full-term deliveries) to better approximate the performance of the DOC/16α-OHP biomarker in a general population. Third, we infer an association between alterations in the DOC/16α-OHP ratio and the HPA phenotype although this is not formally tested. Our data analysis revealed a pattern of altered mineralocorticoid/glucocorticoid concentrations that plausibly influences downstream markers associated with preterm labor. However, we did not perform a global assessment of molecules previously associated with the HPA phenotype and fetal health, such as CRH or DOC sulfate, respectively. In addition, as the study was not designed to do so, we were not able to survey women with a validated tool to assess maternal psychosocial stressors to determine if these stressors are associated with altered progesterone metabolism. Future validation studies will also seek to determine if CRH or DOC sulfate, in combination with other measured steroid metabolites, may be able to predict a woman’s risk for preterm birth. In addition, mechanistic studies are needed to formally link the DOC/16α-OHP ratio to changes in CRH activity.

Longitudinal changes in the maternal–fetal HPA axis have been shown to predispose a pregnant woman to sPTD (Sandman *et al*., 2006). This study has demonstrated that quantification of progesterone-derived molecules as early as the late first trimester may provide insight into which pregnancies are at risk for very preterm delivery (<32 weeks). The DOC/16α-OHP ratio appears to be a particularly promising tool for risk stratification of women early in pregnancy based on our findings. We believe that DOC/16α-OHP is an early biomarker for a mineralocorticoid/glucocorticoid imbalance that influences CRH and the HPA phenotype of sPTD. An ‘abnormal’ DOC/16α-OHP ratio may define a subset of women susceptible to environmental or infectious insults culminating in sPTD. More work is needed to understand the mechanisms linking DOC and 16α-OHP to sPTD; this insight may provide an opportunity to develop targeted therapeutic strategies to improve clinical care. Further studies are needed to validate these findings and create a tool for early identification of pregnancies at risk for very preterm delivery.

## Authors’ roles

A.S.P. contributed to the design of the study and the analysis of the data and drafted the article. N.W.G., S.D.D. and D.M.H. contributed to the acquisition of laboratory and clinical data. C.A.G. made substantial contribution to interpretation and presentation of the data. All authors made contributions to drafting and revising the article critically for important intellectual content. All authors approved the final version of the article to be published.

## Funding

Funding from the Office of the Vice Chancellor for Research at IUPUI, ‘Funding Opportunities for Research Commercialization and Economic Success (FORCES) grant’. Samples obtained from the Building Blocks Pregnancy Biobank at Indiana University School of Medicine were used in this study. We thank contributors, participants, and those who provided funding for the Biobank, including the IUPUI Signature Center grant to PREGMED, The Indiana University Center for Pharmacogenetics and Therapeutics Research in Maternal and Child Health, the Lilly Endowment, Inc. Indiana Genomics Initiative and the Department of OB/GYN.

## Conflict of interest

A.S.P. and C.A.G. are affiliated with Nixxi, a biotech company developing risk stratification tools for preterm birth.
